# Genome-Wide Identification and Evolutionary Analysis of *Sarcocystis neurona* Protein Kinases

**DOI:** 10.3390/pathogens6010012

**Published:** 2017-03-21

**Authors:** Edwin K. Murungi, Henry M. Kariithi

**Affiliations:** 1 Department of Biochemistry and Molecular Biology, Egerton University, P.O. Box 536, 20115 Njoro, Kenya; 2 Biotechnology Research Institute, Kenya Agricultural and Livestock Research Organization, P.O. Box 57811, Kaptagat Rd, Loresho, 00200 Nairobi, Kenya; henry.kariithi@kalro.org

**Keywords:** *Sarcocystis neurona*, EPM, apicomplexans, phylogeny, homology modeling

## Abstract

The apicomplexan parasite *Sarcocystis neurona* causes equine protozoal myeloencephalitis (EPM), a degenerative neurological disease of horses. Due to its host range expansion, *S. neurona* is an emerging threat that requires close monitoring. In apicomplexans, protein kinases (PKs) have been implicated in a myriad of critical functions, such as host cell invasion, cell cycle progression and host immune response evasion. Here, we used various bioinformatics methods to define the kinome of *S. neurona* and phylogenetic relatedness of its PKs to other apicomplexans. We identified 97 putative PKs clustering within the various eukaryotic kinase groups. Although containing the universally-conserved PKA (AGC group), *S. neurona* kinome was devoid of PKB and PKC. Moreover, the kinome contains the six-conserved apicomplexan CDPKs (CAMK group). Several OPK atypical kinases, including ROPKs 19A, 27, 30, 33, 35 and 37 were identified. Notably, *S. neurona* is devoid of the virulence-associated ROPKs 5, 6, 18 and 38, as well as the Alpha and RIO kinases. Two out of the three *S. neurona* CK1 enzymes had high sequence similarities to *Toxoplasma gondii* TgCK1-α and TgCK1-β and the *Plasmodium* PfCK1. Further experimental studies on the *S. neurona* putative PKs identified in this study are required to validate the functional roles of the PKs and to understand their involvement in mechanisms that regulate various cellular processes and host-parasite interactions. Given the essentiality of apicomplexan PKs in the survival of apicomplexans, the current study offers a platform for future development of novel therapeutics for EPM, for instance via application of PK inhibitors to block parasite invasion and development in their host.

## 1. Introduction

Equine protozoal myeloencephalitis (EPM) is an infectious, progressive, degenerative neurological disease of horses caused by the apicomplexan parasite, *Sarcocystis neurona* [1]. To complete its life cycle, this heteroxenous parasite requires a reservoir host (i.e., opossums; *Didelphis virginiana*, *Didelphis albiventris*) and an aberrant (horses) or intermediate host (cats, skunks, raccoons and sea otters) [2]. Opossums become infected upon ingestion of sarcocysts containing hundreds of bradyzoites. The bradyzoites undergo gametogony and sporulate into mature oocysts that are then shed in the feces. After ingestion by the intermediate or aberrant hosts, the oocysts transform into the environmentally-resistant sporozoites that chronically parasitize the neural and inflammatory cells of the host’s central nervous system (CNS). Clinical EPM symptoms depend on the part of the CNS that is parasitized and in general results in abnormal gait, dysphagia and muscle atrophy in affected horses [3].

The intracellular nature of *S. neurona* and its ability to evade the host’s immune surveillance [4] makes EPM treatment expensive, lengthy and challenging. Traditionally, clinical treatment of EPM involved inhibitors of folate synthesis and metabolism (sulfonamides/pyrimethamine combination) over a prolonged period [5]. More recently, triazines derivatives (diclazuril, ponazuril) that target the parasite’s apicoplast [6], nitazoxanide, a pyruvate:ferredoxin oxidoreductase analogue that inhibits the parasite’s anaerobic metabolism [7], and anti-inflammatory agents and immune stimulants [8] have been used with variable success in eliminating clinical signs. Despite the availability of these drugs, EPM treatment is complicated by the emergence of drug-resistance (due to intermittent or periodic treatments), cost of therapies and drug toxicity and infection relapses due to re-growth of residual parasites after the treatment regimes [2]. As such, the discovery and development of novel therapeutics for EPM is imperative.

To successfully invade the host cells, apicomplexans utilize three specialized exocytic organelles (micronemes, rhoptries and dense-granules) [9]. The microneme is used for host cell recognition, binding, penetration and gliding along the cytoskeletal structures. Rhoptry proteins are discharged into the host cell during parasite internalization and are crucial in the formation of the parasitophorous vacuoles (PVs). Developing zoites contain non-pedunculated condensing vesicles that synthesize and package inactive rhoptry proteins, which are proteolytically activated when the rhoptry contents are condensed [10]. The PVs facilitate parasite development by allowing nutrient transport from the host cell and by blocking lysosomal fusion, which would otherwise kill the parasites [11]. Upon internalization, zoites use the dense-granules to remodel the PVs into functionally-active organelles.

The proliferation and differentiation of apicomplexans are influenced by protein kinases (PKs) that are involved in the invasion and modification of host cell structure and function. Generally, PKs can be classified into the conventional (“typical”) eukaryotic PK (ePK) and “atypical” PK (aPK) superfamilies [12,13,14]. Based on the controlled vocabulary of Hanks et al.’s [12,15] classification scheme, there are eight ePK families. These include PKs A, G and C (AGCs), calmodulin/calcium-dependent PKs (CAMKs), CMGC (including cyclin-dependent kinases (CDKs), mitogen-activated protein kinases (MAP kinases), glycogen synthase kinases (GSK) and CDK-like kinases), casein kinase 1 (CK1), “sterile-phenotype” kinases (STEs), receptor guanylate cyclase (RGC), tyrosine kinases (TKs), tyrosine kinase-like kinases (TKLs) and the “other PKs” sub-family (OPKs) [16]. The aPK superfamily consist of the Alpha-kinases, pyruvate dehydrogenase kinases (PDHK), phosphatidylinositol 3-kinase-related kinases (PIKK) and right open reading frame (RIO) kinases [17]. Although generally lacking or having limited sequence similarity to the ePKs and constituting small families in all organisms, some aPKs are homologous to catalytically-active PKs [12].

Several kinomes have been characterized in various organisms [14], including yeast, fruit fly, roundworms and human [18]. In Apicomplexans, the kinome of the malaria parasite, *Plasmodium falciparum*, was initially reported to contain 85 typical ePKs, which clustered into several groups including five of the major ePKs (i.e., CK1, TKL, CMGC, CAMK and AGC), but was devoid of STEs and TKs [18]. Subsequent studies on the *Plasmodium* kinome resulted in the identification of more PKs and PK-like proteins, adding up to 99 PKs [19,20]. However, despite the diverse repertoire of the *Plasmodium* ePKs, reverse genetics studies revealed that over 30% of the kinases are nonessential for the parasite’s asexual blood-stage development; only three of the 12 ePKs required for *Plasmodium* transmission in vivo have been conclusively demonstrated to be essential for the parasite’s asexual development [21]. Kinomes of a dozen other apicomplexan species have been reported, notable of which are *Toxoplasma*, *Cryptosporidium* and *Babesia* species [17]. Talevich et al. [22] recently classified ePKs into 17 genomes in Apicomplexa (*Coccidia*, *Piroplasmida* and *Haemosporida* species). The Rhoptry kinases (ROPKs) and pseudokinases in some coccidian genomes (*Toxoplasma gondii*, *Neospora caninum*, *Eimeria tenella* and portions of *S. neurona*) have been recently catalogued into 42 subfamilies [23]. Overall, at least 65 orthologous PK groups amongst the 12 kinomes described in the apicomplexans are shared with other alveolates and/or metazoans [17,24].

Each of the PK families has vital roles in parasite’s survival. For instance, PfPK-B (AGC family), PfTKL3 (TKL family) and four of the seven CDPKs (CAMK family) are required by *Plasmodium* parasites to complete their asexual cycle [25,26,27]. In a recent study, deletion of TgCK1α (CK1 family) resulted in defective replication of *T. gondii* in vitro [28]. The *P. falciparum* CKL and SRPK1 (CMGC family) complement each other in the regulation of mRNA splicing [29]. Since apicomplexans lack typical MAPK cascades, the STEs are not well studied. However, in the parasites that do have the MAPK pathways, STEs are essential for parasite growth (e.g., in the human parasites, *Schistosoma mansoni* [30]). Further, for parasites without the conventional MAPK cascades, PKs may activate the signaling pathways, for instance the *Plasmodium* Pfnek-1/3 [31,32]. It should however be noted that the activation of *Plasmodium* MAPK via Pfnek-1/3-mediated phosphorylation has only been demonstrated in vitro; there is no sufficient evidence of MAPK signaling in vivo in the parasite. Finally, some of the notable OPKs include aurora kinases, rhoptry kinases (ROPKs) and parasite-specific eukaryotic initiation factor-2 (elF2) kinases (elF2K), which are important in parasite virulence and differentiation [23,33,34].

Here, we used a genome-wide approach to define the kinome of *S. neurona* and determined the relatedness of the putative PKs to those reported in other apicomplexans. Defining the *S. neurona* kinome is not only important in providing insights into the parasite biology, but also identification of potential novel drug targets that can be used to clear chronic *S. neurona* infections and reduce parasite survival.

## 2. Results

### 2.1. Sarcocystis neurona Encodes 97 Putative Kinases

To date, at least 15 apicomplexan genomes (coccidians, gregarines, hemosporidians and piroplasmids) have either been fully sequenced or partially annotated [24]. In the current study, we conducted an exhaustive genome-wide search of the newly-sequenced *S. neurona* genome [35], and identified 97 putative PKs (Table 1). The identified PKs contained the characteristic PK (IPR000719) or PK-like (IPR011009) domains and three conserved amino acids constituting the catalytic triad (Lys30, Asp125, Asp143). The PKs had sizes ranging between 152 and 6544 amino acids and relative molecular weights of between 15.94 and 671.51 kDa. The majority of the PKs had an isoelectric point (pI) greater than 7.0, implying that the PKs have low turnover rates, since in general, acidic proteins are thought to be degraded more rapidly than neutral or basic proteins [36].

Assignment of *S. neurona* PK groups was accomplished by sequence clustering using Blast2GO [37] and by BLASTp searches in the Kinase database [14]. Out of the eleven known PK groups [12,13,14], *S. neurona* PKs segregated into the AGC (*n* = 9), CAMK (*n* = 20), CK1 (*n* = 3), CMGC (*n* = 19), STE (*n* = 2), TKL (*n* = 6), aPK (*n* = 7) and OPK (*n* = 31) (Table 1). Apart from the 31 OPKs that do not fit into the major kinase groups, the CAMK and CMGC groups, whose members are essential for the parasite’s host cell invasion [38] and differentiation (via cell-cycle regulation) [39], respectively, had the highest number of PKs, underlying the importance of these processes in the parasite. Unlike in some parasites, such as *P. falciparum* that lack STEs [18], *S. neurona* contains STE PKs.

#### 2.1.1. The AGC Group

The numbers of apicomplexan AGCs range from four (in *Babesia bovis*) to 15 (in *T. gondii*) [17]. Based on our Blast2GO annotations and BLASTp homology searches against the kinome database, five out of the nine *S. neurona* AGCs (SRCN_3339, SRCN_3990, SRCN_5165, SRCN_5610 and SRCN_1312) were homologs to the universally-conserved PKAs that are found in *N. caninum* and *T. gondii* (see Table 1). The PKAs are essential for the completion of schizogony (asexual reproduction) in *Plasmodium* parasites [40]. Further, *S. neurona* contains a putative PKG (SRCN_4518), which shows high homology (92%) to the *T. gondii* TgPKG1 (Table 1); PKGs are essential in apicomplexans [41].

#### 2.1.2. The CAMK Group

CAMKs form the second-largest apicomplexan PKs (after OPKs). Apicomplexan kinomes constitute varying numbers of CAMKs, which range from seven (in *B. bovis*) to 29 (in *T. gondii*) [17]. The most important CAMK family is the CDPK, which appeared to constitute almost 50% of *S. neurona* putative CAMKs (see Table 1). In terms of homologies, the *S. neurona* kinome contained orthologs to the *T. gondii* CDPK1 (SCRN_3314), CDPK2B (SCRN_2165), CDPK3 (SCRN_3701), CDPK4 (SCRN_6606), CDPK5 (SCRN_3583), CDPK6 (SCRN_3011), CDPK7 (SCRN_6597) and CDPK8 (SCRN_5948). Other CDPK orthologs were to the *N. caninum* CDPK2 (SCRN_4390) and *Hammondia hammondi* CDPK9 (SCRN_5812) (Table 1). Inhibition of TgCDPK1 has been shown to disrupt the motility, host cell invasion and egress of *T. gondii* [43]. Owing to the absence of mammalian CDPK homologs, the identification of a relatively large number of CDPK homologs in *S. neurona* could be utilized in the rational design of anti-parasitic therapeutics.

#### 2.1.3. The CK1 Group

It is notable that *S. neurona* putatively encodes for three CK1 enzymes. Apart from *T. gondii* and some alveolates (e.g., *Cryptosporidium hominis* and *Cryptosporidium parvum*, important causative agents of diarrhea in children), which have three and two CK1 enzymes, respectively, most apicomplexans possess a single CK1 enzyme [17]. Two of the three *S. neurona* putative CK1 (SRCN_3445 and SRCN_4645) showed high sequence similarity (>90%) to the *T. gondii* TME49_040640 (TgCK1-α) and TGME49_089320 (TgCK1-β), respectively (Table 1). Inhibition of CK1 showed potential for anti-parasitic interventions in *T. gondii* [44]. CK1 is critical for the asexual proliferation of the *Plasmodium* parasites and is expressed in all of the life-cycle stages of the parasite [45]. Three putative *S. neurona* CK1 had significant sequence similarity to the *P. falciparum* PfCK1, i.e., 74% (SRCN_3445), 65% (SRCN_4587) and 56% (SRCN_4645) (data not shown).

#### 2.1.4. The CMGC Group

The CMGC is the largest PK group in apicomplexans; CMGC numbers range from 15 in *B. bovis* to 23 in *Plasmodium vivax* [17], which is within the range we identified in the *S. neurona* kinome in our study (i.e., 19 CMGCs; see Table 1). Notable of these were the two GSK homologs (SRCN_1731 and SRCN_1732). This finding is similar to what has been observed in *Plasmodium* parasites in which two GSK-3 enzymes have been reported, both of which are essential for the parasite [46]. Homology searches showed considerable sequence similarity (51% and 41% for SRCN_1731 and SRCN_1732, respectively) to the PfGSK-3 enzymes (data not shown). Notably, eight of the 19 CMGCs in *S. neurona* were CDKs, including CDK7 (SRCN_4674, SRCN_2759 and SRCN_761), CDK10 (SRCN_895) and CDK11 (SRCN_977). Available data show that CDKs are essential in *P. falciparum* [24]. We also identified two putative MAPK homologs (SRCN_4209 and SRCN_5365) and ERK7 (SRCN_6472) (see Table 1), a result that is comparable to the two MAPKs in the kinome of *P. falciparum* [17].

#### 2.1.5. The OPK Group

The apicomplexan-specific OPKs are a tight cluster of PKs without clear relation to any of the other major PK groups. Notable of these are ROPKs, which have high sequence divergence and have been thought to be largely restricted to *T. gondii* [47], which has a total of 34 members spread in over 40 distinct sub-families [23]. Although their diversification in apicomplexans is poorly understood, some ROPKs are key virulence factors in *T. gondii* [23]. At least nine putative ROPKs could be identified in *S. neurona*, including ROPK19A (SRCN_6184), ROP27 (SRCN_3247), ROP30 (SRCN_2076), ROP33 (SRCN_7082 and SRCN_7086), ROP35 (SRCN_2183, SRCN_2123, SRCN_7083 and SRCN_4410) and ROP37 (SRCN_7084), implying that the ROPKs are not restricted to *T. gondii*. Although largely presumed to be inactive, ROPKs are implicated in the regulation of the host transcription [47], and their presence in *S. neurona* may support the hypothesis that the ROPKs have a unique activation mechanisms in their regulatory functions that facilitate apicomplexan pathogenesis [24,48]. Other notable OPKs included two parasite-specific eukaryotic initiation factor-2 (elF2) kinases (elF2K-C (SRCN_1606) and elF2K-B (SRCN_4503)), four NEKs (SRCN_4528, SRCN_2630, SRCN_286 and SRCN_3151) and four ULKs (SRCN_3444, SRCN_3669, SRCN_6812 and SRCN_6157) (Table 1). The elF2Ks are conserved in apicomplexans and are important for the induction of parasite differentiation into the bradyzoites cysts, which are clinically important [34].

#### 2.1.6. The STE Group

The STEs are poorly represented in apicomplexans, and although most apicomplexans have one or two STE genes per genome, some parasites, such as *C. parvum*, are reported to harbor up to six STEs [17,20]. Our results suggest that *S. neurona* has at least one putative STE (Table 1). STEs are thought to function in MAPK pathway cascades despite the fact that this pathway is absent in apicomplexans. The small repertoire of apicomplexan STEs is in contrast to that reported in other parasites, such as trypanosomatids, in which these enzymes regulate the length of the flagella [49].

#### 2.1.7. The TKL Group

Apicomplexans harbor a maximum of seven TKL-coding genes, which makes it notable that we identified six putative TKLs in *S. neurona* (Table 1). Reverse genetics studies have demonstrated that some of the conserved TKLs, for instance PfTKL3, are essential for the asexual *Plasmodium* proliferation [27], thereby a potential drug target. Two of the six *S. neurona* putative TKLs had considerable sequence similarities to the *Plasmodium* TKLs, including SRCN_3466 (36% similar to *Plasmodiuim malariae* TKL1) and SCRN_1435 (49% similar to *Plasmodium ovale* TKL3) (data not shown).

#### 2.1.8. The aPK Group

The aPKs have been detected in apicomplexan parasites, such as *P. falciparum* [17,18] and *T. gondii*, which has at least four genes thought to encode these enzymes, the products of which are hypothesized to be part of the ovoid mitochondrial cytoplasmic (OMC) complex [50], a composite assembly of organelles observed only in growing tachyzoites of *T. gondii*. An exhaustive search of the *S. neurona* proteome revealed four putative PIKKs (SRCN_3988, SRCN_6464, SRCN_6465, SRCN_1259) and one PDHK (SRCN_1743) (Table 1). Whereas PIKKs have been identified in at least 12 apicomplexan kinomes, PDHK seem to have been identified only in the *T. gondii* kinome [17]. Our analyses of the putative *S. neurona* PKs did not yield any homologs of the Alpha and RIO kinases, implying that these PKs are absent from the kinome of this parasite; RIO kinases have been reported in *P. falciparum* [17,18], as well as in the kinomes of other apicomplexans including *C. parvum*, *T. gondii* and *B. bovis* [17].

### 2.2. Evolution of S. neurona Protein Kinases

**Figure 1:**
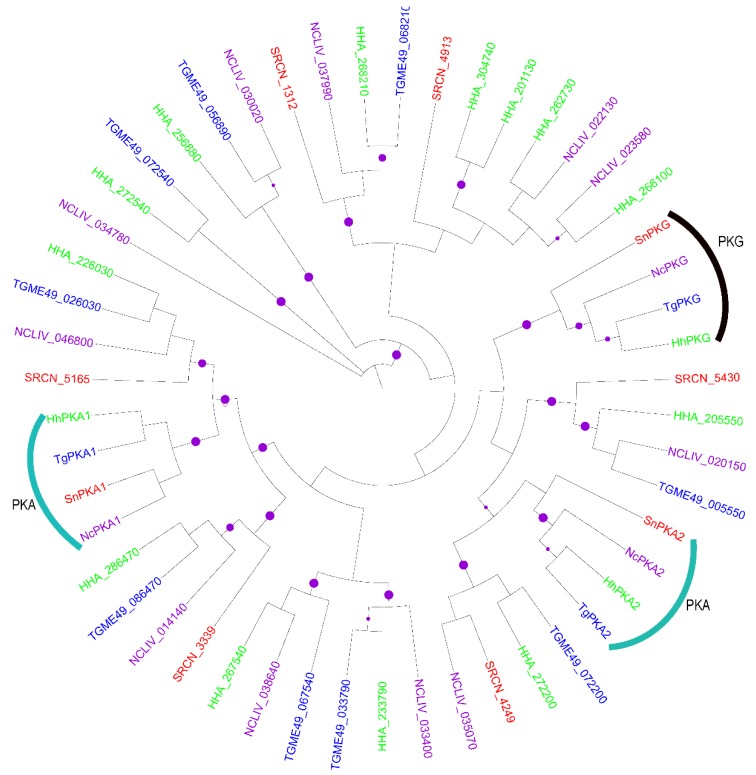
Mid-point rooted maximum likelihood (ML) phylogenetic tree of apicomplexan AGCs. The terminal branches are color-coded for AGCs in the kinomes of *Sarcocystis neurona* (SRCN; red), *Toxoplasma gondii*, ME-49 strain (TGME49; blue), *Hammondia hammondi* (HHA; green) and *Neospora caninum*, Liverpool strain (NCLIV; purple). A solid purple circle on a branch indicates bootstrap support greater than 70. The phylogenetic tree was inferred from a multiple sequence alignment using PhyML with the Le and Gascuel (LG) amino acid substitution model and the gamma model of substitution rate heterogeneity. The tree image was rendered with iTOL.

We investigated the evolutionary relationships among the various *S. neurona* PK groups and their homologs in related apicomplexans. Our analysis revealed valuable insights into the biology of these organisms. The kinome of *S. neurona* is comprised of slightly fewer AGCs (*n* = 9) compared to the kinomes of *T. gondii* (*n* = 11), *N. caninum* (*n* = 13) and *H. hammondi* (*n* = 15). In general, the phylogenetic clustering of the *S. neurona* AGCs mirrored the homologies of these enzymes to those of the three apicomplexans used in this study (Figure 1; compare with Table 1). Sequence analysis of *S. neurona* AGCs revealed significant divergence with only ~30% sequence similarity amongst members of this group. Two *S. neurona* AGCs SRCN_5610 (SnPKA1) and SRCN_3990 (SnPKA2) clearly cluster with *T. gondii* PKAs TGME49_028420 and TGME49_015670 [51] (Figure 1). Moreover, SRCN_5610 shares high (~60%) full length sequence identity with its ortholog, TgPKA1. It is also notable that the single putative PKG (SRCN_4518) distinctly clustered with its *T. gondii* ortholog, TGME49_111360 (TgPKG) (Figure 1). It has recently been shown that *P. falciparum* PKG acts as a signaling hub that plays a central role in a number of core parasite processes [52].

In addition to the kinase domain, SnPKA1, SRCN_3339, SRCN_5165 and SRCN_4518 possess the AGC-kinase C-terminal domain, which contains two of the three conserved phosphorylation sites in AGCs (data not shown). These conserved sites serve as phosphorylation-regulated switches in the control of both intra- and inter-molecular interactions [53]. Like *T. gondii*, *S. neurona* lacks PKB and PKC. However, *S. neurona* contains a putative phosphoinositide-dependent kinase-1, PDPK1 (SRCN_1312), that clusters with the *T. gondii* PDPK1 (TGME49_268210) [51].

**Figure 2:**
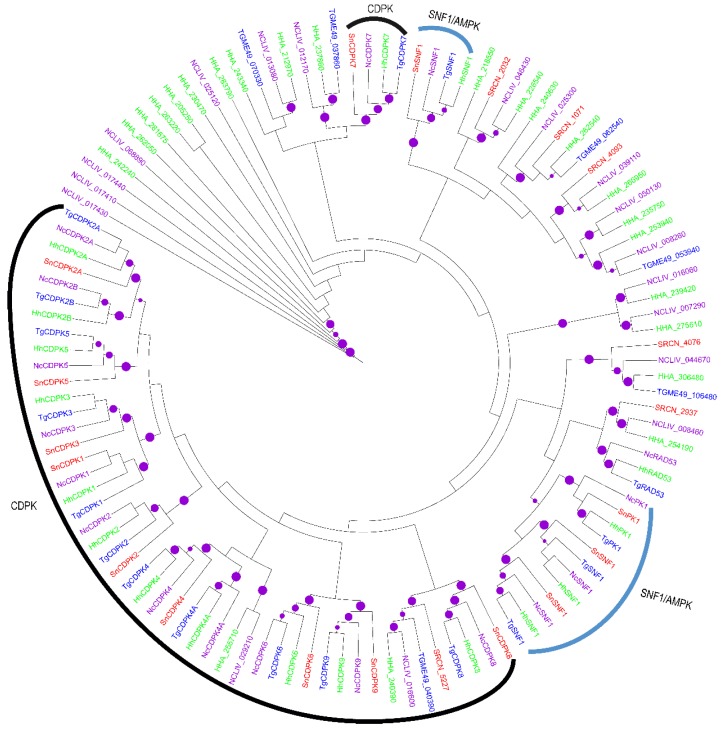
Mid-point rooted ML phylogenetic tree of apicomplexan CAMKs. The terminal branches are color-coded for AGCs in the kinomes of *S. neurona* (SRCN; red), *T. gondii*, ME-49 strain (TGME49; blue), *H. hammondi* (HHA; green) and *N. caninum*, Liverpool strain (NCLIV; purple). A solid purple circle on a branch indicates bootstrap support greater than 70. The phylogenetic tree was inferred from a multiple sequence alignment using PhyML with the LG amino acid substitution model and the gamma model of substitution rate heterogeneity. The tree image was rendered with iTOL.

Despite the absence of PKC in *S. neurona*, CAMK family members were identified, which perhaps underscores the importance of Ca^2+^ regulation in this apicomplexan parasite. The majority of the identified *S. neurona* CAMKs segregated with their orthologs in *T. gondii*, *N. caninum* and *H. hammondi* in clades with robust bootstraps (Figure 2), thus validating the annotation of the CAMKs. Amongst the CAMKs, SRCN_2544 clustered with *T. gondii* PK1 (TGME049_243500) of the AMPK/SNF1 sub-family. There were also three additional SNF1 members in *S. neurona* (SRCN_5410, SRCN_4815 and SRCN_2257), which clustered with *T. gondii* TGME49_315190, TGME49_233905 and TGME49_291050, respectively.

Based on the clustering with *T. gondii* CDPK orthologs, 10 *S. neurona* CDPKs, including CDPK1 (SRCN_3314), CDPK2 (SRCN_4390), CDPK2A (SRCN_2165), CDPK3 (SRCN_3701), CDPK4 (SRCN_6606), CDPK5 (SRCN_3583), CDPK6 (SRCN_3011), CDPK7 (SRCN_6597), CDPK8 (SRCN_5948) and CDPK9 (SRCN_5812), were identified (Figure 2). This result implies that *S. neurona* has potentially lost at least two CDPKs (compared to the 12 CDPK that have been reported in *T. gondii* [26]). The possible loss notwithstanding, *S. neurona* contained the six CDPKs that are expressed and are well-conserved in most apicomplexans (i.e., CDPK1, CDPK3, CDPK4, CDPK5, CDPK6 and CDPK7) [54]. Sequence analysis revealed that, like in other apicomplexans, all identified *S. neurona* CDPKs except SnCDPK7 contained both a kinase domain and a Ca^2+^-binding domain known as the EF-hand domain [26]. Like its *T. gondii* ortholog, TgCDPK7) SnCDPK7 contains a pleckstrin-homology (PH) domain just upstream of its PK domain [54]. The domain architecture in CDPKs is such that kinase activity is stimulated upon Ca^2+^-binding. Putative additional *S. neurona* CDPKs include SRCN_5227, which falls within the CDPK cluster and segregates with TGME49_040390 that is annotated as a CDPK and SRCN_4076 that clusters with TGME49_106480, also annotated as a CDPK (Figure 2).

**Figure 3:**
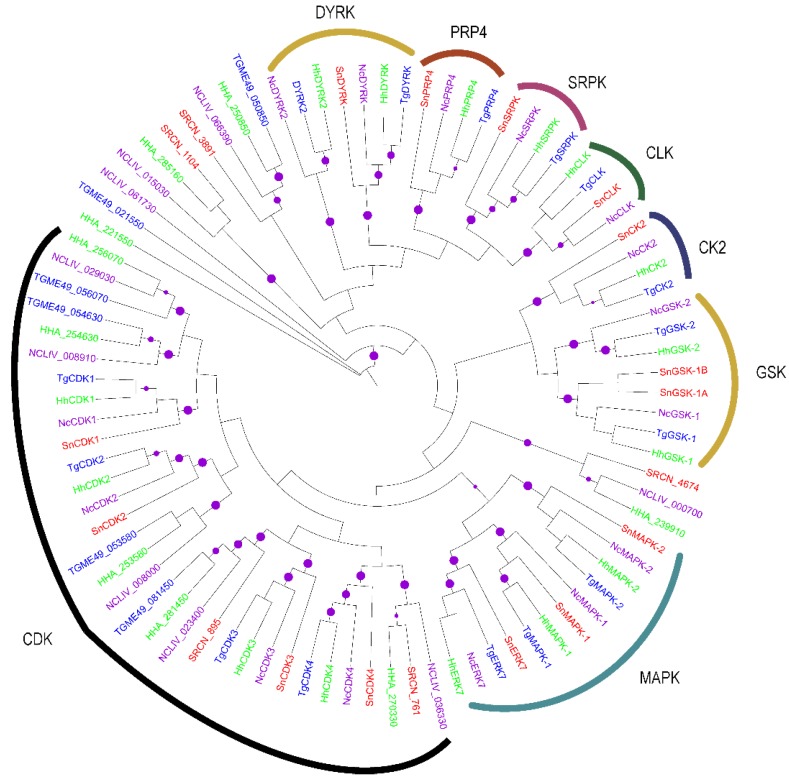
Mid-point rooted ML phylogenetic tree of apicomplexan CMGCs. The terminal branches are color-coded for AGCs in the kinomes of *S. neurona* (SRCN; red), *T. gondii*, ME-49 strain (TGME49; blue), *H. hammondi* (HHA; green) and *N. caninum*, Liverpool strain (NCLIV; purple). A solid purple circle on a branch indicates bootstrap support greater than 70. The phylogenetic tree was inferred from a multiple sequence alignment using PhyML with the LG amino acid substitution model and the gamma model of substitution rate heterogeneity. The tree image was rendered with iTOL.

The majority of the putative CMGCs identified in *S. neurona* clustered with robust bootstrap support values with the conserved CMGCs in *T. gondii*, *N. caninum* and *H. hammondi* (Figure 3). Based on the segregation of the CMGC kinases, four *S. neurona* CDK1 (SRCN_4801), CDK2 (SRCN_2759), CDK3 (SRCN_977) and CDK4 (SRCN_6346) were identified (Figure 3). CDKs are amongst the main molecular switches that regulate cell cycle progression in apicomplexan parasites [55]. Additional *S. neurona* CMGC kinases identified include SRPK (SRCN_1236), CLK (SRCN_1479), PRP4 (SRCN_2845), DYRK (SRCN_1611), GSK-1A (SRCN_1731), GSK-1B (SRCN_1732), CK2 (SRCN_6427), ERK7 (SRCN_6472), MAPK-2 (SRCN_5365) and MAPK-1 (SRCN_4209), all of which fall in orthologous clades with robust bootstrap values. SRPK, CLK and PRP4 kinases most likely have crucial roles in parasite survival given their involvement in cycle-regulatory regulation [56,57,58], potentially via alternative mRNA splicing. Inhibition of PfCLK-mediated SR protein phosphorylation impaired blood stage replication and malaria transmission in *Plasmodium* [59]. The DYRK is implicated in a myriad of cell cycle functions that make this PK, hence an attractive drug target [60]. Other drug targets include MAPKs, which regulate diverse cellular functions, such as tissue morphogenesis, cytoskeletal rearrangements, proliferation, differentiation, survival, immune responses and adaptation/stress-responses [61]. The *S. neurona* putative MAPK-1 (SRCN_4209) ortholog in *T. gondii* (TgMAPK-1) is a virulence factor that alters IFN-γ-mediated control of *Toxoplasma* tachyzoite proliferation by manipulating IFN-γ-mediated nitric oxide synthase (iNOS) and NO generation [62].

**Figure 4:**
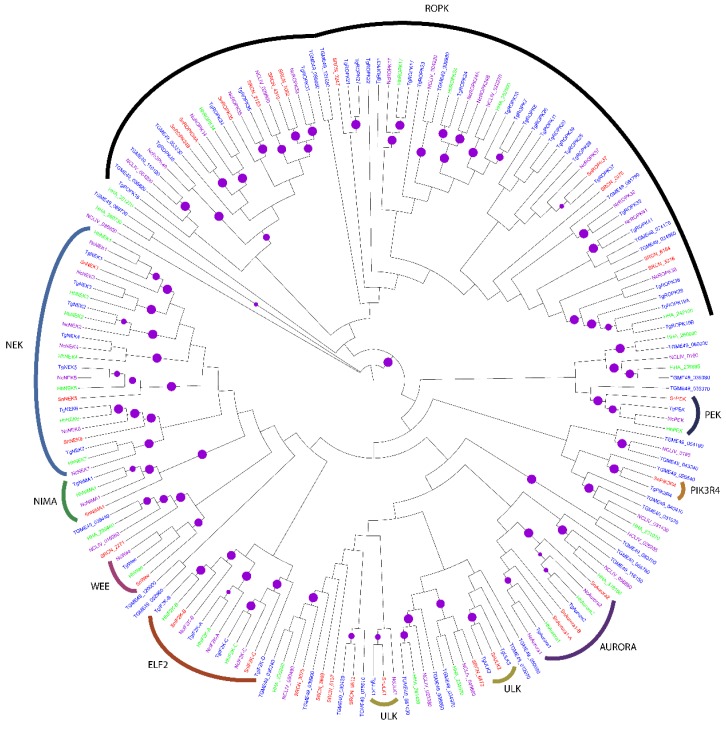
Mid-point rooted ML phylogenetic tree of apicomplexan OPKs. The terminal branches are color-coded for AGCs in the kinomes of *S. neurona* (SRCN; red), *T. gondii*, ME-49 strain (TGME49; blue), *H. hammondi* (HHA; green) and *N. caninum*, Liverpool strain (NCLIV; purple). A solid purple circle on a branch indicates bootstrap support greater than 70. The phylogenetic tree was inferred from a multiple sequence alignment using PhyML with the LG amino acid substitution model and the gamma model of substitution rate heterogeneity. The tree image was rendered with iTOL.

In the OPK family, SRCN_4528, SRCN_2630 and SRCN_3151 are putative NEKs given their clustering with *T. gondii* NEK kinases TgNEK1 (TGME49_319700), TgNEK6 (TGME49_294260) and TgNEK5 (TGME49_018400), respectively (Figure 4). *S. neurona* has two putative ULK kinases SRCN_108 (SnULK2) and SRCN_3444 (SnULK1) that cluster with their *T. gondii* orthologs TgULK1 (TGME49_235750) and TgULK2 (TGME49_240630), respectively). *S. neurona* contains three putative Aurora kinases; SRCN_2404 (SnAurora1-A), SRCN_2403 (SnAurora1-B) and SRCN_3417 (SnAurora2) that cluster with *T. gondii* aurora kinases, TgAurora1 (TGME49_118770) and TgAurora2 (TGME49_003010) (Figure 4). *S. neurona* also contains a putative Wee kinase, SnWee (SRCN_286) that clusters with *T. gondii* Wee kinase, TgWee (TGME49_273690), as well as a NIMA kinase, SnNIMA1 (SRCN_5943), that clusters with TgNIMA1 (TGME49_292140). NIMA-related kinases are implicated in cell cycle control. Like its apicomplexan relatives *T. gondii* and *P. falciparum*, *S. neurona* contains tyrosine-kinase-like (TKL) kinase, SRCN_6572, that clusters with TGME49_234970. Moreover, *S. neurona* contains two elF2 kinases, SnIF2K-B (SRCN_4503) and SnIF2K-C (SRCN_1606) (Figure 4). SRCN_4503 shows 74% sequence similarity to the *T. gondii* TgCatPRC2 [IF2K-B] (Table 1).

The clustering of putative *S. neurona* ROPKs was also notable. For instance, SRCN_7084 (SnROPK37) clustered with *T. gondii* TgROPK37 (TGME49_094560), implying that it is a ROPK37 (Figure 4). SRCN_7082 and SRCN_4310 cluster with *T. gondii* TgROPK33 (TGME49_001130), suggesting that they are isoforms of ROPK33. Annotations in Table 1 indicate that SRCN_4310 is a putative ROP33 (39% sequence similarity to *H. hammondi* ROP33). Moreover, SRCN_2183 (SnROPK35) clusters with TgROPK35 (TGME49_104740). Other putative *S. neurona* ROPKs include SRCN_7083 (SnROPK34A) and SRCN_4410 (SnROPK34B) that cluster with TgROP34 (TGME49_040090) (Figure 4). SnROPK34A and SnROPK34B are potentially duplicated forms of TgROPK34.

Moreover, SRCN_3142 (SnPIK3R4) segregates with *T. gondii* TgPIK3R4 (TGME49_018550). NEKs are involved in cell cycle regulation, while Aurora kinases play pivotal roles in endodyogeny, duplication rate and parasite virulence [33]. Taken together, the presence of a variety of ROPKs in *S. neurona* is interesting given the fact that in *T. gondii*, ROPKs are key virulence factors [63].

## 3. Discussion

The kinomes of apicomplexans range from 35 PKs (in *B. bovis*) to 135 PKs (in *T. gondii*) [24]. We identified a total of 97 putative PKs in the kinome of *S. neurona*, compared to the PKs reported in the kinomes of *P. falciparum* (*n* = 99), *T. gondii* (*n* = 135), *N. caninum* (*n* = 130) and *H. hammondi* (*n* = 124) [17,19]. Although the total number of *S. neurona* PKs appeared markedly reduced compared to that of its close coccidian relatives (*T. gondii* and *N. caninum* [23]), taken as a percentage of total genome size, the proportion of *S. neurona* PKs is comparable to the 2% observed in humans [13] and other coccidians [23]. The contraction of the *S. neurona* kinome could be attributed to genome compaction, which occasionally offsets lineage-specific expansions of specific gene families. Notably, genome contraction is a common mode of genomic evolution in intracellular parasites, including apicomplexans [64,65]. As such, the evolution of PKs may be in tandem to the overall genomic adaptive strategies of these parasites.

Using a hierarchical scheme based on the major PK groups, the *S. neurona* kinases could be classified and phylogenetically clustered into the various PK families. A complement of nine putative AGC kinases was identified in *S. neurona*, which is reduced compared with that of *T. gondii*, *N. caninum* and *H. hammondi*. Despite this potential gene loss, seven of the nine AGCs (SRCN_5165, SRCN_5610, SRCN_3339, SRCN_4249, SRCN_3990, SRCN_5430 and SRCN_1312) had orthologs in *T. gondii*, *N. caninum* and *H. hammondi*. In agreement with the observation that PKA is conserved in apicomplexans [23], two PKAs (SRCN_5610 and SRCN_3990) were identified in *S. neurona.* In *T. gondii*, increases in cytosolic cAMP levels activate PKA to trigger the developmental switch from the rapidly proliferating tachyzoites to the quiescent bradyzoites [66]. Additionally, two other *S. neurona* AGCs (SRCN_5165 and SRCN_3339) were putative PKAs given that they contained the characteristic *GxGxxG* motif found in PKA [51]. Notably, based on orthology, *S. neurona* contains a single putative PKG (SRCN_4518) that distinctly clustered with *T. gondii* PKG (TGME49_111360).

In apicomplexans, CAMKs modulate the intracellular Ca^2+^ concentration, which in turn regulates vital processes, such as host-cell invasion, protein secretion and parasite differentiation. We identified four potential AMPK/SNF1 family members (SRCN_2544, SRCN_5410, SRCN_4815 and SRCN_2257). The AMP-activated PK cascade acts as a metabolic sensor that monitors cellular AMP and ATP levels and is activated by an elevation of the AMP:ATP ratio. Further, we identified 12 putative CDPKs in the *S. neurona*, including CDPK1 (SRCN_3314), CDPK2 (SRCN_4390), CDPK2A (SRCN_2165), CDPK3 (SRCN_3701), CDPK4 (SRCN_6606), CDPK5 (SRCN_3583), CDPK6 (SRCN_3011), CDPK7 (SRCN_6597), CDPK8 (SRCN_5948), CDPK9 (SRCN_5812), SRCN_5227 and SRCN_4076. Compared to the 12 CDPKs reported in *T. gondii* [26], it appears that *S. neurona* had all six well-conserved apicomplexan CDPKs (CDPK1, CDPK3, CDPK4, CDPK5, CDPK6 and CDPK7), which provide a link between Ca^2+^ signaling and parasite differentiation, motility, invasion and egress [54]. In *T. gondii*, downregulation of CDPK1 interfered with parasite motility, host cell invasion and egress [43], while disruption of CDPK3 caused defective parasite egress [67]. Further, the essentiality of CDPK6 and CDPK7 in *T. gondii* has recently been demonstrated [68]. Indeed, TgCDPK1 has been targeted for the development of new drugs for toxoplasmosis [69]. Sequence analysis revealed that, similar to other apicomplexans, all identified *S. neurona* CDPKs except CDPK7 (SRCN_6597) contain both a PK domain and an EF-hand (Ca^2+^-binding) domain [26]. Similar to its *T. gondii* ortholog, TGME49_028750 (TgCDPK7), the *S. neurona* CDPK7 (SRCN_6597) contains a pleckstrin-homology (PH) domain just upstream of its PK domain [54]; the domain architecture is such that kinase activity is stimulated upon Ca^2+^ binding. Moreover, our phylogeny provided clues of possible gene duplications giving rise to SRCN_3990 and SRCN_3011, as well as SRCN_4093 and SRCN_1071. Interestingly, based on phylogenetic analysis, *S. neurona* probably contains four (SRCN_5227, SRCN_1071, SRCN_4093 and SRCN_3011) species-specific CAMKs.

The CMGCs, comprising CDKs, MAPKs, GSKs and CLKs, coordinate a wide range of cellular functions in different species. By both annotations and phylogenetic analyses, we identified four putative CDKs sub-family members; CDK5 (SRCN_4801 and SRCN_6346), CDK7 (SRCN_2759, SRCN_4674 and SRCN_761), CDK10 (SRCN_895) and CDK11 (SRCN_977). The finding of CDKs in *S. neurona* suggests that this parasite’s cell cycle regulation could be CDK-dependent and perhaps similar to that of higher eukaryotes [70]. The identification of three putative MAPKs (SRCN_4209, SRCN_6472 and SRCN_5365) in *S. neurona* points to the existence of MAPK regulated transduction pathway(s) in this pathogen. Similar to its *T. gondii* ortholog (TgMAPK1), which is a p38α MAPK homolog [71], SRCN_4209 may be potentially involved in parasite proliferation/stage differentiation, stress response and manipulation of the host immunity to enhance virulence. On the other hand, SRCN_6472 and SRCN_5365 may augment the roles of SRCN_4209 in the parasite. In *T. gondii*, MAPK1/ERK7 is involved in intracellular proliferation [72]. We also identified two putative GSKs (SRCN_1731 and SRCN_1732). A genome-wide gene knockout approach in *P. falciparum* demonstrated PfGSK-3 to be critical for schizogony of the parasite [19]. Other *S. neurona* CMGC kinases identified include CLK (SRCN_1479), PRP4 (SRCN_2845), DYRK (SRCN_1611) CK2 (SRCN_6427) and SRPK (SRCN_1236).

ROPKs are secreted by *T. gondii* into the host cell and play roles in adhesion, motility and manipulation of immune responses [73]. We identified 11 putative ROPK sub-family members in the *S. neurona* kinome, i.e., ROPK37 (SRCN_7084), ROPK35 (SRCN_2183), ROPK33A (SRCN_7082), ROPK33B (SRCN_4310), ROPK34A (SRCN_7083), ROPK34B (SRCN_4410), SRCN_6184, SRCN_3216, SRCN_2076, SRCN_3247 and SRCN_2123. It has been recently demonstrated that *T. gondii* ROP21 and ROP27 play a role in a constitutive pathway based on their localization in the PV and cyst matrix [74]. However, the *S. neurona* putative ROPs 21 and 27 could not be clearly delineated in our clustering. Moreover, *T. gondii* ROP35 has been shown to play a crucial role in chronic infection [75]. Although the *S. neurona* genome is more than twice the size of other coccidians whose genomes were sequenced so far (e.g., *Toxoplasma* and *Neospora*), it has a considerably reduced number of ROPKs that nevertheless may have vital roles in the parasite’s virulence. Specifically, *S. neurona* is devoid of ROP5, ROP16, ROP18 and ROP38, which have been shown to confer virulence and alter the host’s cellular signaling pathways [72]. Putatively therefore, *S. neurona* ROPKs may have multiple roles in the survival of the parasite. In the search of drug targets against *S. neurona*, the reduced ROPKs with possible multiple roles and absent in the vertebrate host are thus attractive candidates.

## 4. Conclusions and Future Perspectives

The kinome of *S. neurona* contains members of the major classes of PKs, including AGC, CMGC, GSK, CAMK, CK, TKL, aPKs and several PKs in the OPK family. Similar to other apicomplexans, *S. neurona* kinome is devoid of PKC, the TKs, Alpha kinases, as well as RIO kinases. Further, the *S. neurona* kinome harbors two putative MAPK homologs, a finding that is similar to some apicomplexans, such as *P. falciparum*. *S. neurona* kinome also lacks some of the ROPKs that have been implicated in the virulence of *T. gondii*. Given the central roles played by PKs in the regulation of the host-parasite interactions and in the facilitation of the parasite proliferation and differentiation, delineation of the *S. neurona* kinome offers a platform for future development of efficacious drugs for EPM, for instance via parasite transmission blocking vaccine against the parasites (specific inhibition of the parasite’s PKs). This approach is made possible by the differences between parasite and host PK homologs [76]. Zhang et al. [77] reviewed the applications and the progress made in the targeting of specific PKs as antimalarial drugs against *Plasmodium* parasites. Proof of principle of this approach has been demonstrated by the inhibition of human PKs using chemical ligands to treat cancers and other diseases [78,79]. Recently, Ojo et al. [80] provided evidence that PKs can be targeted for rationally-designed drugs that can potently inhibit the growth of *S. neurona*. The technology is available and approved for therapeutic intervention, thus offering a unique prospect of repurposing chemical ligands to manage *S. neurona* infections [81]. It is however important to note that experimental validations are required to validate the *S. neurona* putative PKs to facilitate the development of anti-parasitic interventions. A potential approach is the application of genetically-encoded sensors to identify inhibitors of important parasite signaling pathways.

## 5. Materials and Methods

### 5.1. Genome-Wide Identification of Putative S. neurona PKs

The predicted *S. neurona* proteome was downloaded from the Toxoplasma Genomics Resource database (Release 28; Version May 2016) [42]. A hidden Markov model (HMM) profile of signature PK domains obtained from the Kinomer database v 1.0 [82] was used to search for *S. neurona* kinases using HMMER v 3.1b2 [83]. The sequences having PK domain (IPR011009) or PK-like domain (IPR000719) were considered as putative kinases. Annotation of the putative kinase sequences was performed by BLASTp search against the non-redundant (nr)-NCBI protein and UniProtKB/Swiss-Prot databases at an *e*-value of ≤10^−6^. The identified *S. neurona* putative PKs were subsequently classified by BLASTp interrogations into the KinBase [84]. Gene ontology (GO) mapping was performed using Blast2GO v 4.0.7 [37]. The molecular weight (Mw) and isoelectric point (pI) were obtained using the ExPASy compute pI/Mw tool [85]. Motifs analysis was performed with the MEME Suite v 4.11.2 [86]. The parameters were as follows: number of repetitions, any; maximum numbers of motifs, 30; and the optimum motif widths, between 6 and 200 residues.

### 5.2. Phylogenetic Analysis

Phylogenetic trees were constructed to decipher the orthologous and paralogous relationships of *S. neurona* kinases. Protein kinase domains from putative *S. neurona* kinase groups were extracted and aligned with protein kinase domains from their homologs in *T. gondii* [42], *H. hammondi* and *N. caninum* using MUSCLE [87]. The alignments were subsequently manually edited in Jalview [88] for curation of alignment to remove uncertain regions due to gaps and poor alignment. Phylogenetic reconstruction was undertaken using the maximum likelihood program PhyML 3.0 [89] and RAxML v 8.0 [90] and the Bayesian inference program MrBAYES v 3.2 [91]. For PhyML, the LG substitution model was selected assuming an estimated proportion of invariant sites and four gamma-distributed rate categories to account for rate heterogeneity across sites. The gamma shape parameter was estimated directly from the data. The robustness of internal branches was evaluated using 100 bootstraps. MrBayes was run for 5,000,000 generations with two runs and four chains in parallel and a burn-in of 25%. Obtained trees were rendered with the Interactive Tree of Life server (iTOL) [92].

## Figures and Tables

**Table 1:** Description of the 97 putative protein kinases (PKs) identified in the kinome of *Sarcocystis neurona*. The putative PKs could be classified into eight groups. The amino acid coordinates of the conserved PK domains in the protein sequences and the PK homologies to other apicomplexan PKs are shown in Columns 7–12.

Description of the Putative Protein Kinases (PKs) in the Genome of *S. neurona*							Description of Protein Kinase (PK) Homologies (BLASTp)				
Protein ID ^a^	Sequence Annotations; Description ^b^	Family; (Subfamily) ^c^	Length (aa)	pI	MW (kDa)	PK Domain Coordinates	Sequence Name; (Apicomplexan)	Bit Score	*E*-Value	Identity (%)	Accession Number
**1. Kinase Group AGC (Protein kinases A (PKA), G (PKG) and C (PKC) families)**											
SRCN_1312	AGC kinase	3-phosphoinositide dependent PK-1 (PDK1)	903	5.52	101.13	137–481	PDPK; (*T. gondii* RUB)	417	6.00 × 10^−135^	59	KFG61374.1
SRCN_3339	AGC kinase	PKA	1428	8.85	147.52	1102–1417	Putative AGC kinase; (*N. caninum* L)	611	0.0	81	CEL65574.1
SRCN_4249	AGC kinase	Nuclear dbf2-related (NDR)	152	8.91	17.47	6–141	Putative AGC kinase; (*N. caninum* L)	219	2.00 × 10^−68^	76	XP_003883757.1
SRCN_4518	PK G AGC kinase family member PKG	Ciliate-E2	425	5.83	48.78	97–399	AGC kinase TgPKG1; (*T. gondii* ME49)	830	0.0	92	EPR61116.1
SRCN_3990	cAMP-dependent kinase	CAMKL; (MELK)	1907	9.55	217.55	782–1634	cAMP-dependent protein kinase (*T. gondii* VEG)	75.9	1 × 10^−12^	30	ESS31194.1
SRCN_5165	cAMP-dependent PK, catalytic chain	PKA	343	8.99	39.36	20–338	AGC kinase; (*T. gondii* ARI)	425	3.00 × 10^−150^	90	KYF43224.1
SRCN_4913	Putative PK	PKD	2330	6.42	244.64	1093–1737	Putative PK; (*E. tenella*)	107	9.00 × 10^−22^	60	XP_013228294.1
SRCN_5430	AGC kinase	Ribosomal protein S6 Kinases (RSK; (p70))	1378	5.54	139.28	824–1344	AGC kinase; (*T. gondii* MAS)	223	3.00 × 10^−60^	59	KFH07588.1
SRCN_5610	cAMP-dependent PK, catalytic chain	PKA	333	9.00	37.96	12–318	cAMP-dependent PK, catalytic subunit; (*T. gondii* ME49)	641	0.0	92	XP_002366464.1
**2. Kinase Group calcium (Ca^2+^)-/calmodulin-regulated kinases (CAMK)**											
SRCN_1071	Ca^2+^-dependent kinase	CAMK1	1495	9.47	152.66	1085–1401	Putative PK; (*T. gondii* VEG)	188	1.00 × 10^−47^	67	ESS31884.1
SRCN_2032	Putative PK	Ciliate-C1	297	6.22	33.26	15–297	PK; (*H. hammondi*)	424	1.00 × 10^−146^	68	XP_008882026.1
SRCN_2165	Ca^2+^-dependent kinase CDPK2B	CDPK	692	7.33	75.65	101–401	Ca^2+^-dependent PK CDPK2A; (*T. gondii* ARI)	674	0.0	90	KYF44522.1
SRCN_2257	Histone kinase	CAMKL; (AMP-activated protein kinase (AMPK))	1800	8.98	187.71	1159–1448	Putative CAM kinase, SNF1 family; (*E. acervulina*)	377	7.00 × 10^−105^	64	XP_013252246.1
SRCN_2544	CAM SNF1 AMK1 family	CAMKL;(AMPK-regulated kinase novel kinase (NUAK))	333	5.71	37.85	62–333	CAM kinase, SNF1/AMK1 family ToxPK1; (*N. caninum* L)	480	9.00 × 10^−167^	79	XP_003882065.1
SRCN_2937	Ca^2+^-signaling kinase MARK	CAMKL; (microtubule affinity regulating kinase (MARK))	278	9.62	31.29	1–250	Putative Ca^2+^ signaling PK MARK; (*T. gondii* GT1)	356	5.00 × 10^−124^	72	EPR59053.1
SRCN_3011	Calmodulin-dependent PK (CAM) CDPK6	CDPK	1435	9.25	154.44	1238–1435	Cdpk kinase domain; (*T. gondii*)	179	6.00 × 10^−49^	75	3IS5_A
SRCN_3314	A Chain crystal Structure of TgCDPK1 with inhibitor bound	CDPK1	519	5.99	58.89	37–335	Calmodulin-domain PK 1; (*T. gondii*)	536	0.0	97	3MA6_A
SRCN_3583	Ca^2+^-dependent kinase CDPK5	CDPK	454	6.09	50.38	35–308	Ca^2+^-dependent PK CDPK5; (*T. gondii* ARI)	776	0.0	89	KYF43137.1
SRCN_3701	Ca^2+^-dependent kinase CDPK3	CDPK	560	5.91	62.07	77–362	Ca^2+^-dependent Kinase; (*T. gondii*)	493	1.00 × 10^−172^	87	3DXN_A
SRCN_4076	CAM CDPK family	CDPK	1701	5.93	181.42	1079–1663	CAM kinase, CDPK family; (*H. hammondi*)	239	2.00 × 10^−62^	69	XP_008884897.1
SRCN_4093	PK	CAMKL; (AMP-activated protein kinase (AMPK))	1155	9.02	118.33	1–261	Putative atypical MEK-related kinase; (*N. caninum* L)	222	1.00 × 10^−59^	70	XP_003880869.1
SRCN_4390	Ca^2+^-dependent kinase CDPK2	CDPK	790	6.24	85.47	280–556	Ca^2+^-dependent PK, related; (*N. caninum* L)	1130	0.0	74	XP_003884321.1
SRCN_4815	Histone kinase (partial)	CAMKL; (AMP-activated protein kinase (AMPK))	711	6.53	75.98	1–314	SNF1-related PK catalytic-α KIN10, 5 AMP-activated PK; (*N. caninum* L)	568	0.0	48	CEL67550.1
SRCN_5227	CAM CDPK CDPK8-like	CDPK	2748	8.98	282.12	796–885	Putative CAM kinase, CDPK family; (*N. caninum* L)	117	3.00 × 10^−24^	59	XP_003881901.1
SRCN_5410	Calmodulin-dependent PK (CAM-SNF1 family)	CAMK1	467	8.94	52.04	168–446	CAM kinase, SNF1 family; (*H. hammondi*)	431	5.00 × 10^−134^	78	XP_008883430.1
SRCN_5812	Ca^2+^-dependent kinase CDPK9	CDPK	760	8.37	84.23	254–573	Ca^2+^-dependent PK CDPK9; (*H. hammondi*)	1139	0.0	81	XP_008889286.1
SRCN_5948	Ca^2+^-dependent kinase CDPK8	CDPK	3298	7.11	345.85	208–860	EF-hand domain-containing protein; (*T. gondii* ME49)	114	1.00 × 10^−23^	58	XP_002368547.1
SRCN_6597	Ca^2+^ dependent kinase CDPK7	CAMK1	1374	9.09	138.28	365–623	PK-PH domain-containing protein; (*T. gondii* ME49)	813	0.0	80	XP_002366487.1
SRCN_6606	Ca^2+^-dependent kinase CDPK4	CDPK	1632	9.42	170.93	813–1236	Ca^2+^-dependent PK; (*T. gondii*)	731	0.0	58	CAD32376.2
**3. Kinase Group casein kinase 1 (cell kinase 1)**											
SRCN_3445	Casein kinase I	CK1-D	323	9.34	37.65	6–290	Casein kinase 1; (*T. gondii* ME49)	603	0.0	94	XP_002366683.1
SRCN_4587	Casein kinase I	CK1-D	137	7.78	15.94	1–137	Casein kinase I; (*H. hammondi*)	226	3.00 × 10^−71^	81	XP_008883809.1
SRCN_4645	Casein kinase I	CK1-D	229	9.51	25.66	38–229	Casein kinase I; (*T. gondii* GAB2-2007-GAL-DOM2)	259	1.00 × 10^−86^	79	KFG42638.1
**4. Kinase Group CMGC (including cyclin-dependent kinases, mitogen-activated PKs, glycogen synthase kinases and CDK-like kinases)**											
SRCN_1104	Cyclin-dependent kinase family 5	Ca^2+^-dependent PK-L (CDKL)	372	9.23	42.71	1–318	Cyclin-dependent kinase family 5 protein; (*H. hammondi*)	490	2.00 × 10^−173^	76	XP_008884207.1
SRCN_1236	Cell-cycle-associated kinase (SRPK)	Serine-arginine rich PK (SRPK)	2911	5.37	302.90	713–1837	PK; (*T. gondii* ME49)	476	9.00 × 10^−141^	76	XP_002369401.1
SRCN_1479	CMGC Lammer	CLK	748	10.23	79.09	485–748	Cell-cycle-associated PK CLK; (*T. gondii* FOU)	288	2.00 × 10^−81^	74	KFG33061.1
SRCN_1611	CMGC Dual-specificity tyrosine-regulated kinase (DYRK)	DYRK; (DYRKP)	1504	5.86	160.34	551–1498	Cell-cycle-associated PK DYRK; (*T. gondii* VEG)	223	2.00 × 10^−57^	63	ESS33160.1
SRCN_1731	Cell-cycle-associated kinase GSK	Glycogen synthase kinase (GSK)	219	6.59	24.39	1–175	Cell-cycle-associated PK GSK; (*H. hammondi*)	330	2.00 × 10^−112^	82	XP_008887193.1
SRCN_1732	Cell-cycle-associated kinase GSK	Glycogen synthase kinase (GSK)	203	10.78	20.72	86–203	CMGC kinase, GSK family TgPK3; (*E. brunetti*)	114	1.00 × 10^−28^	91	CDJ46527.1
SRCN_2759	Cell-cycle-associated kinase partial	Ca^2+^-dependent PK (CDK); (CRK7)	1122	6.14	118.67	541–1122	Cell-cycle-associated PK CDK; (*T. gondii* VAND)	118	1.00 × 10^−25^	79	KFH12036.1
SRCN_2845	CMGC DYRK PRP4 kinase	DYRK; (PRP4)	1665	9.76	177.08	1267–1596	Putative PK (CLK3); (*P. malariae*)	330	1.00 × 10^−102^	69	SBS85334.1
SRCN_3891	CMGC kinase	DYRK; (DYRK2)	674	8.85	73.85	399–674	Putative CMGC kinase; (*T. gondii* ME49)	80.1	6.00 × 10^−14^	67	EPT25192.1
SRCN_4209	CMGC MAPK family (ERK) MAPK-1	Mitogen-activated PK (MAPK); (ERK))	2361	6.73	247.71	94–754	CMGC, MAPK/ (ERK) TgMAPK-1; (*E. brunetti*)	137	1.00 × 10^−30^	74	CDJ49492.1
SRCN_4674	Cyclin-dependent kinase	Ca^2+^-dependent PK (CDK); (CDK7)	138	7.80	15.50	1–138	Cyclin-dependent kinase; (*T. gondii* GT1)	108	7.00 × 10^−27^	58	EPR60430.1
SRCN_4801	Cell-cycle-associated kinase	Ca^2+^-dependent PK (CDK); (CDK5)	300	6.08	34.33	1–289	CMGC kinase, CDK family TgPK2; (*N. caninum* L)	576	0.0	91	XP_003885801.1
SRCN_5365	Cell-cycle-associated kinase MAPK	Mitogen-activated PK (MAPK; (ERK))	417	6.77	48.32	7–363	Cell-cycle-associated PK MAPK; (*H. hammondi*)	823	0.0	93	XP_008886907.1
SRCN_6346	Cell-cycle-associated kinase CDK	Ca^2+^-dependent PK (CDK); (CDK5)	690	9.55	80.90	208–603	Putative cell-cycle-associated PK CDK; (*T. gondii* ARI)	390	2.00 × 10^−122^	87	KYF45878.1
SRCN_6427	CMGC CK2 kinase	Cell Kinase 2 (CK2)	1395	10.29	144.86	885–1356	CMGC kinase, CK2 family; (*T. gondii* MAS)	241	6.00 × 10^−73^	98	KFH07655.1
SRCN_6472	Cell-cycle-associated kinase ERK7	Mitogen-activated PK (MAPK; (ERK))	983	9.28	104.95	7–317	Cell-cycle-associated PK ERK7; (*T. gondii* ARI)	647	0.0	81	KYF46268.1
SRCN_761	Cell-cycle-associated kinase	Ca^2+^-dependent PK (CDK); (CDK7)	577	9.34	58.39	144–490	Cell-cycle-associated PK; (*H. hammondi*)	283	3.00 × 10^−88^	68	XP_008882409.1
SRCN_895	Cell-cycle-associated kinase	Ca^2+^-dependent PK (CDK); (CDK10)	340	8.93	38.57	1–307	Cell-cycle-associated PK; (*T. gondii* ARI)	234	6.00 × 10^−75^	76	KYF44017.1
SRCN_977	Cell-cycle-associated kinase CDK	Ca^2+^-dependent PK (CDK); (PITSLRE/CDK11)	1502	7.38	156.35	1114–1429	Cell-cycle-associated PK CDK; (*T. gondii* p89)	454	3.00 × 10^−135^	92	KFG28420.1
**5. Kinase Group ‘Other’ (OPK; i.e., kinases with conventional PK (ePK) domains that do not fit into any of the other major groups of kinases)**											
SRCN_108	Unc-51-like autophagy activating kinase 1 (ULK1)	ULK	343	7.13	38.90	1-223	ULK kinase; (*T. gondii* VAND)	376	2.00 × 10^−130^	75	KFH07419.1
SRCN_1606	eIF2 kinase IF2K-C	PEK; (general control nonderepressible 2 (GCN2))	4034	8.98	406.57	1235–2178	eIF2 kinase IF2K-C; (*T. gondii* VAND)	259	4.00 × 10^−67^	35	KFH07289.1
SRCN_2076	Rhoptry kinase family ROP30	Conserved hypothetical protein	1276	9.18	134.73	812–1260	ROP30 (*T. gondii* VEG)	230	3.00 × 10^−63^	53	CEL76436.1
SRCN_2123	Rhoptry kinase family ROP35	PLK; (PLK-Unclassified)	291	9.30	33.50	53–265	ROP35; (*T. gondii* RUB)	207	2.00 × 10^−61^	43	KFG59037.1
SRCN_3216	Rhoptry kinase family ROP32	CAMK-Unique	523	7.08	57.00	214–520	Putative PK; (*T. gondii* VAND)	167	1.00 × 10^−42^	30	KFH00232.1
SRCN_2183	Rhoptry kinase family ROP35	Aurora-like	226	6.36	25.84	1–212	ROP35; (*T. gondii* VEG)	198	8.00 × 10^−59^	48	ESS33297.1
SRCN_2271	Putative PK (incomplete catalytic triad)	NimA (Never in mitosis gene A)-related Kinase (NEK)	1463	9.02	157.18	437–1145	Putative PK; (*N. caninum* L)	327	5.00 × 10^−90^	68	XP_003881849.1
SRCN_2403	Aurora kinase (incomplete catalytic triad)	PLK; (SAK/Plk4)	778	9.79	79.92	492–778	Putative Aurora kinase; (*N. caninum* L)	127	3.00 × 10^−28^	44	XP_003880644.1
SRCN_2630	NimA related kinase (NEK) family protein	NEK	351	8.70	38.38	1–336	NEK kinase; (*T. gondii* ME49)	242	8.00 × 10^−75^	52	XP_018638598.1
SRCN_286	Wee kinase	Inhibitory regulator of the *RAS*-cAMP (IRA1) kinase suppressor (IKS)	1019	6.20	106.67	598–959	Wee kinase; (*H. hammondi*)	445	5.00 × 10^−141^	58	XP_008882669.1
SRCN_3075	Tyrosine kinase-like (TKL) protein	Numb-associated kinase (NAK)	1571	8.41	164.18	16–500	TKL; (*T. gondii* TgCatPRC2)	138	1.00 × 10^−32^	73	KYK64203.1
SRCN_3142	PIK3R4 kinase-related	Aurora	997	8.72	106.54	548–899	Putative PIK3R4 kinase-related protein; (*N. caninum* L)	449	2.00 × 10^−137^	60	XP_003885774.1
SRCN_3151	NimA related kinase (NEK) family protein	NEK	3186	7.96	318.69	352–656	NEK kinase; (*T. gondii* VEG)	468	7.00 × 10^−131^	73	CEL78174.1
SRCN_3247	Rhoptry kinase family ROP27	Ciliate-D	345	8.94	38.81	23–325	ROP27; (*T. gondii* p89)	163	4.00 × 10^−43^	31	KFG37427.1
SRCN_3417	Aurora kinase	Aurora	438	7.65	48.49	14–289	Aurora kinase; (*T. gondii* TgCatPRC2)	490	3.00 × 10^−155^	76	KYK63669.1
SRCN_3444	Unc-51-like Autophagy activating kinase 1 (ULK1)	ULK	406	6.52	44.69	12–406	ULK kinase; (*T. gondii* RUB)	232	3.00 × 10^−71^	61	KFG59767.1
SRCN_3669	CMGC kinase	ULK	1803	8.41	189.65	736–1200	Putative CMGC kinase; (*N. caninum* L)	624	0.0	62	CEL65030.1
SRCN_4410	Rhoptry kinase family ROP35	PKA-like	204	9.44	23.50	1–166	ROP35; (*H. hammondi*)	107	1.00E × 10^−25^	39	XP_008885989.1
SRCN_4503	eIF2 kinase IF2K-B	PEK; (general control nonderepressible 2 (GCN2))	158	5.76	17.59	1–158	eIF2 kinase IF2K-B (*T. gondii* TgCatPRC2)	149	5.00 × 10^−41^	74	KYK69938.1
SRCN_4528	NimA related kinase (NEK) family protein	NEK	187	8.20	21.23	1–186	NEK kinase; (*H. hammondi*)	177	4.00 × 10^−54^	64	XP_008885186.1
SRCN_2404	Aurora kinase (incomplete catalytic triad)	Serum and glucocorticoid induced Kinase (SGK)	295	8.81	31.42	1–249	Putative Aurora kinase; (*N. caninum* L)	126	7.00 × 10^−31^	43	CEL65223.1
SRCN_5653	PEK kinase	Aurora	626	8.27	60.75	513–626	PEK kinase (*T. gondii* TgCatPRC2)	251	1.00 × 10^−76^	60	KYK62422.1
SRCN_5943	NIMA-related kinase NIMA1	NEK	2842	9.04	295.44	73–383	NIMA-related PK NIMA1; (*T. gondii* MAS)	486	2.00 × 10^−140^	67	KFH05809.1
SRCN_6157	Unc-51-like autophagy activating kinase 1 (ULK1)	ULK	2420	9.38	250.58	1380–1672	ULK kinase (*T. gondii* ME49)	99.8	3 × 10^−21^	38	XP_018635814.1
SRCN_6184	Myosin-light-chain kinase	Ciliate-E2-Unclassified	478	5.42	53.65	177–474	ROP19A (*T. gondii ME49*)	127	3.00 × 10^−30^	27	XP_018637476.1
SRCN_6572	Tyrosine kinase-like (TKL)	ULK	622	6.13	68.80	1–345	TKL; (*T. gondii* VAND)	181	1.00 × 10^−46^	74	KFH00338.1
SRCN_6812	PK	ULK	199	6.74	22.72	1–183	PK; (*H. hammondi*)	172	3.00 × 10^−48^	53	XP_008887491.1
SRCN_7083	Rhoptry kinase family ROP35	PKA-like	262	9.62	30.26	1–242	ROP35; (*H. hammondi*)	127	6.00 × 10^−32^	39	XP_008885989.1
SRCN_4310	Rhoptry kinase family ROP33	Kinase Homologous to SPS1/STE20 (KHS)	1591	9.85	169.63	1265–1578	ROP33; (*H. hammondi*)	306	2.00 × 10^−87^	39	XP_008887632.1
SRCN_7082	Rhoptry kinase family ROP33	Kinase Homologous to SPS1/STE20 (KHS)	403	9.59	45.92	77–390	ROP33 (*T. gondii* p89)	277	3.00 × 10^−89^	40	KFG45248.1
SRCN_7084	Rhoptry kinase family ROP37	Ribosomal protein S6 Kinases (RSK; (RSK))	339	5.41	38.09	19–334	ROP37; (*N. caninum* L)	144	1.00 × 10^−36^	36	CEL64242.1
**6. Kinase Group “Sterile” serine/threonine kinase, or sterile-phenotype kinases (STE)**											
SRCN_1328	Serine threonine kinase	Conserved hypothetical protein	1461	9.29	158.74	559–671	Hypothetical protein, conserved; (*E. maxima*)	88.6	5.00 × 10^−16^	68	XP_013335801.1
SRCN_5172	“Sterile” serine/threonine kinase (STE)	Mammalian Sterile 20-like (MST))	6552	6.14	671.51	3410–4122	STE kinase; (*T. gondii* TgCatPRC2)	412	1.00 × 10^−114^	54	KYK71951.1
**7. Kinase Group Tyrosine Kinase-Like (TKL)**											
SRCN_1435	Tyrosine kinase-like (TKL)	Mixed lineage kinase (MLK); (Leucine Zipper-bearing Kinase (LZK))	3064	8.05	306.74	2540–3060	Tyrosine kinase-like (TKL) protein; (*N. caninum* L)	278	3.00 × 10^−73^	72	CEL64955.1
SRCN_1571	Tyrosine kinase-like (TKL)	Microtubule-associated S/T kinase (MAST)	550	9.76	59.91	135–501	Conserved hypothetical protein; (*E. praecox*)	76.3	6.00 × 10^−13^	53	CDI87140.1
SRCN_3466	Tyrosine kinase-like (TKL)	TKL-Unique	3002	9.87	320.97	2342–2997	Tyrosine kinase-like (TKL) protein; (*H. hammondi*)	202	3.00 × 10^−50^	65	XP_008887506.1
SRCN_3928	Tyrosine kinase-like (TKL)	LISK - LIMK (LIM kinase) and TESK (Testicular protein Kinase); (DD1)	5842	8.78	608.80	3639–4268	Tyrosine kinase-like (TKL) protein; (*T. gondii* TgCatPRC2)	216	2.00 × 10^−61^	79	KYK63216.1
SRCN_4277	Kinase domain-containing protein	TKL-ciliate1	2256	8.43	240.25	1570–2256	Tyrosine kinase-like (TKL) protein; (*N. caninum* L)	204	8.00 × 10^−51^	61	CEL67693.1
SRCN_811	Tyrosine kinase-like (TKL)	TKL-Unique	1099	9.18	119.26	814–1083	Putative tyrosine kinase-like (TKL) protein; (*E. acervulina*)	403	6.00 × 10^−125^	59	XP_013252162.1
**8. Kinase Group Atypical (aPKs)**											
SRCN_3601	Atypical MEK-related kinase	Muscle-associated kinase TRIO	950	7.20	103.14	381–850	Atypical MEK-related kinase; (*T. gondii* GT1)	171	1.00 × 10^−42^	32	EPR62774.1
SRCN_5962	Atypical MEK-related kinase	Rho-associated protein kinase (ROCK)-like	805	5.01	87.71	525–805	Atypical MEK-related kinase; (*H. hammondi*)	127	4.00 × 10^−29^	56	XP_008884362.1
SRCN_3988	Phosphatidylinositol 3-/4-kinase (PI3K)	Atypical/PIKK/ATM	4251	5.95	440.5	3671–3772	PI3K, ; (*H. hammondi*)	268	1 × 10^−69^	55	XP_008886631.1
SRCN_6465	Phosphatidylinositol 3-/4-kinase (PI3K)	Atypical PIKK/ATM	207	5.2	23.24	5-122	Phosphatidylinositol 4-kinase, partial; (*T. gondii* p89)	272	4 × 10^−93^	96	KFG28404.1
SRCN_1259	Phosphatidylinositol 3-/4-kinase (PI3K)	Atypical/PIKK/FRAP	1362	9.56	142.85	1140–1311	PI3K; (*T. gondii* MAS)	317	3 × 10^−94^	76	KFH10008.1
SRCN_6464	Phosphatidylinositol 3-/4-kinase (PI3K)	No hits found	2108	8.95	209.68	1626–1722	Phosphatidylinositol 3-4-kinase; (*T. gondii* p89)	244	1 × 10^−63^	66	KFG28409.1
SRCN_1743	Pyruvate dehydrogenase kinase	Atypical/PDHK/BCKDK	930	6.50	101.41	291–426	PDHK, isoenzyme-2; (*N. caninum* L)	458	6 × 10^−146^	45	CEL70411.1

^a^ The protein sequences and their corresponding identified were obtained from the Toxoplasma Genomics Resource database (Release 28; Version May 2016) [42]; ^b^ the descriptions of the protein sequence are based on BLASTp annotations using Blast2GO (see the text for details); ^c^ the kinase classification is based on BLASTp on the kinase database.
